# Minimally invasive esophagectomy drives a decade-long decline in upper mediastinal recurrence after esophageal cancer surgery: a real-world, single-center cohort study

**DOI:** 10.3389/fonc.2025.1711115

**Published:** 2025-11-19

**Authors:** Shifa Zhang, Anhao Hu, Jiuhe Sun, Yutao Wei, Jishan Zhang, Kaize Zhong, Haibo Cai, Gongchao Wang

**Affiliations:** 1 Department of Thoracic Surgery, Shandong Provincial Hospital, Shandong University, Jinan, Shandong, China; 2 Department of Thoracic Surgery, Jining NO.1 People’s Hospital, Affiliated Jining NO.1 People’s Hospital of Jining Medical University, Jining, Shandong, China; 3 Department of Clinical Medicine, Jining Medical University, Jining, Shandong, China

**Keywords:** minimally invasive, esophageal cancer, recurrence, lymph nodes, surgery, neoadjuvant therapy

## Abstract

**Background:**

Surgery is a cornerstone in the treatment of esophageal cancer, yet high postoperative recurrence rates remain a significant challenge. This study aimed to retrospectively examine the trends in postoperative recurrence rates of esophageal cancer over the past decade and identify factors influencing these trends.

**Methods:**

Data from 918 esophageal cancer patients who underwent surgery at Jining First People’s Hospital between January 2013 and May 2021 were analyzed. The follow-up period ranged from 32 to 98 months. Monthly recurrence rates were calculated to observe trends over time. Surgical techniques included various open and minimally invasive approaches. Follow-up assessments involved regular clinical evaluations and imaging studies. Statistical analyses were performed using SPSS, GraphPad Prism, and R software to identify factors influencing recurrence.

**Results:**

A total of 918 patients were included, with 224 experiencing local recurrence. The local recurrence rate showed a significant year-over-year decline (p = 0.000), primarily driven by a reduction in upper mediastinal lymph node recurrence (r = 0.4086, p = 0.0006). In contrast, distant recurrence rates did not exhibit a significant trend. Univariate and multivariate analyses identified several factors influencing upper mediastinal lymph node recurrence, including surgical method, alcohol consumption, number of lymph nodes dissected, and pTNM stage. Minimally invasive esophagectomy (MIE) was found to be an independent factor associated with a reduced monthly recurrence rate in the upper mediastinum (r = -0.3009, p = 0.0134).

**Conclusions:**

The study demonstrated a consistent decline in local recurrence rates over the past decade, particularly in upper mediastinal lymph nodes. The adoption of minimally invasive surgical techniques, improvements in perioperative management, and the evolving role of neoadjuvant therapy likely contributed to this trend.

## Introduction

Surgery is a crucial cornerstone in the treatment of esophageal cancer. Two large randomized controlled trials (1, 2) have demonstrated that the combination of preoperative neoadjuvant chemoradiotherapy (CRT) with surgery is superior to surgery alone. Additionally, a substantial cohort study by Sivesh K. Kamarajah et al. (3) indicates that patients who underwent surgery following neoadjuvant CRT exhibited a significant survival benefit compared to those who received only radical chemoradiation. The data showed a median overall survival (OS) of 18.5 months for the surgery group versus 3.2 months for the non-surgery group, with a hazard ratio (HR) of 0.60 and a 95% confidence interval (CI) of 0.53-0.57 (P < 0.001). These findings underscore the necessity of integrating preoperative neoadjuvant CRT with surgical intervention for advanced esophageal cancer. Consequently, the combination of preoperative neoadjuvant chemoradiotherapy (CRT) and surgery continues to be the most effective treatment for locally advanced esophageal squamous cell carcinoma (ESCC).

Several studies have indicated that the total recurrence rate after radical esophageal surgery ranges from 38% to 52.4%, with local recurrence rates between 32.6% and 49%. Among patients with positive lymph nodes, the rate of distant metastasis is notably high, ranging from 19.8% to 61.3% (4–6). Additionally, even for esophageal cancer patients who undergo neoadjuvant chemoradiotherapy prior to surgery, the 5-year survival rate remains relatively low, at 39% to 47% (1, 7). The reasons behind these outcomes are multifaceted and complex, highlighting an urgent need to better understand the patterns of recurrence and to explore effective response strategies and potential new directions.

Over the past decade, the evolution of minimally invasive thoracic surgery, notably video-assisted thoracoscopy, has revolutionized the surgical approach for esophageal cancer (8). This shift has moved from traditional left thoracotomy to thoracic laparoscopy, expanding the scope of lymph node dissection from the second field to include even the third field, with a marked increase in the number of lymph nodes dissected (9). Concurrently, preoperative neoadjuvant chemoradiotherapy has been validated to enhance esophageal cancer prognosis. The growing number of clinical studies (10–12) on neoadjuvant immunochemotherapy further highlights its potential to improve outcomes.

Additionally, enhanced awareness of esophageal cancer has led to more frequent early screening and diagnosis, increasing the adoption of preoperative neoadjuvant therapies that minimally impact surgical procedures, and more standardized postoperative adjuvant treatments. These advancements hold promise for improving patient survival and prognosis. Despite these progresses, the specific impact on recurrence rates post-surgery remains unclear, as does the strategy for effectively reducing these rates.

Therefore, the objective of this study is to retrospectively examine the trends in postoperative recurrence rates of esophageal cancer over the past decade. Specifically, it aims to determine whether there has been a consistent year-by-year decrease in recurrence rates and identify which types of relapses have shown significant declines. This analysis will also assess which treatments and interventions have contributed to the reduction in recurrence rates and investigate potential therapies that could further decrease these rates. The findings will provide a reliable foundation for developing future treatment strategies for esophageal cancer.

## Methods

### Research subjects

**Figure 1:**
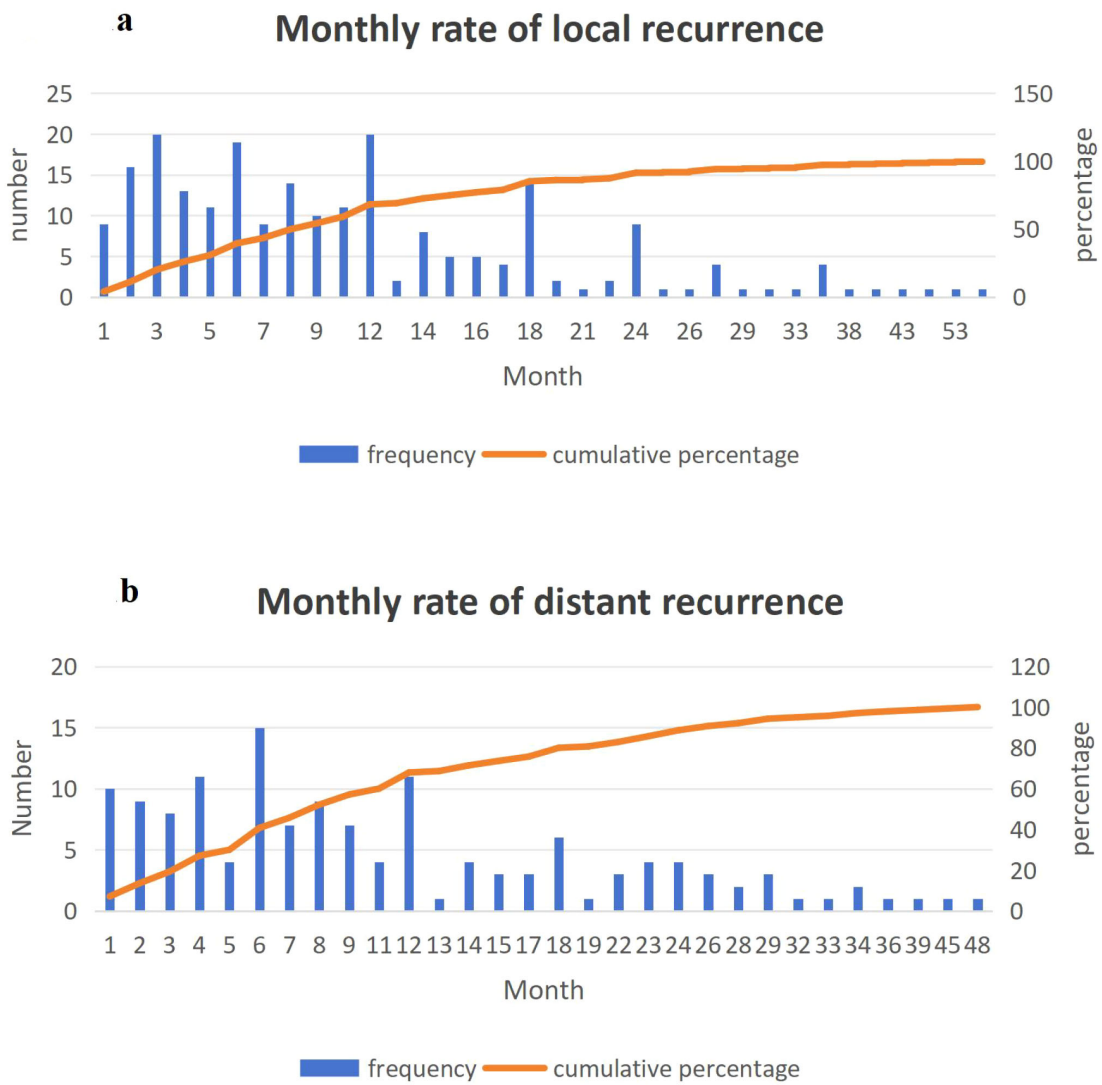
**(A)** Local recurrence of esophageal cancer occurred in 95% of cases within 32 months post-surgery. **(B)** Distant recurrence of esophageal cancer occurred in 95% of cases within 32 months post-surgery.

We collected data from 918 esophageal squamous cell carcinoma patients at Jining First People’s Hospital between January 2013 and May 2021, with a follow-up period ranging from 32 to 98 months. To analyze changes in the recurrence rate over time, we used months as the basic unit to calculate the monthly recurrence rate following esophageal cancer surgery from 2013 to 2021, **Figure 1**. This approach enabled us to observe trends in recurrence and investigate the underlying influencing factors. All patients had a preoperative pathological diagnosis of esophageal malignancies. The inclusion criteria were as follows: (1) Patients diagnosed with esophageal malignancies who underwent surgical resection; (2) Pathological stages ranging from stage I to IIIB; Exclusion Criteria: Exclusion of patients lacking follow-up data.

### Surgical techniques

The surgical approaches utilized in this study encompass a variety of procedures including: McKeown surgery, which involves a transabdominal approach, transright thoracic access, and neck anastomosis; Ivor Lewis surgery, characterized by transabdominal and transright thoracic approaches; and their minimally invasive counterparts. Additionally, the study included mediastinoscopy combined with laparoscopic partial resection of the esophagus and cervical esophagogastrostomy, which also involves transabdominal and neck surgeries. Advanced techniques such as robot-assisted minimally invasive McKeown and Ivor Lewis surgeries were also employed, along with partial esophagectomy through left chest or thoracoabdominal incisions, concluding with esophagogastric (or colonic or jejunal) anastomosis at the chest or neck level.

### Follow-up protocol

Follow-up schedules after esophageal cancer surgery are structured as follows: within the first two years, patients are reviewed every 3 to 6 months; from years three to five, reviews are conducted every 6 to 12 months; and annual reviews are conducted after five years. Follow-up methods include telephone calls, outpatient visits, and scheduled reviews. During follow-up, all patients underwent serial chest computed tomography, combined cervical and abdominal ultrasonography, and repeated serum tumor-marker assessments to enable dynamic surveillance for disease recurrence, and when necessary, gastroscopy, and PET-CT scans.

The endpoint of follow-up is to determine if the patient has experienced a relapse, including local recurrence or distant metastatic recurrence. Local recurrence encompasses regional lymph node recurrence and recurrences at the anastomotic site or gastric body. Regional lymph node recurrence includes recurrent laryngeal, upper mediastinal (encompassing upper circumesophageal and pararyngeal), mid-mediastinal (including middle paraesophageal, subprotuberous, thoracic paraaortic, and left parabronchial nodes), cervical, and celiac nodes (such as left gastric paraarterial, Group 8 lymph nodes, splenic, and hepatic nodes). Distant metastases may involve the brain, liver, bones, lungs, kidneys, adrenals, thoracic structures, and chest or abdominal wall nodules. Recurrence is defined as occurring 0 months post-surgery in patients who undergo intraoperative thoracotomy exploration. The location of the first detected recurrence is recorded as the primary site, and multiple recurrences identified within one month are classified as simultaneous recurrences.

### Definition of minimum follow-up

The minimum follow-up interval was fixed at 32 months because cumulative-incidence analyses demonstrated that 95% of all local and distant recurrences occurred within this postoperative window (**Figures 1A, B**).

### Statistical analysis

Statistical processing of data was conducted using SPSS 27 software, with frequencies weighted by case. Quantitative data are presented as mean ± standard deviation, and comparisons between groups were performed using the T-test. Categorical data are expressed as frequencies (percentages), and the chi-square test was used for intergroup comparisons. Univariate and multivariate analyses were carried out to identify factors influencing progression-free survival (PFS). The chi-square test was utilized for univariate analysis, while binary logistic regression was applied for multivariate analysis. Additionally, parts of the data were analyzed using GraphPad Prism 8 and R software version 3.6.3. The Kaplan-Meier method was employed to generate the disease-free survival (DFS) curves, and parallel logistic regression analyses were conducted. A p-value of less than 0.05 was considered to indicate statistical significance.

## Results

### General clinical characteristics

**Figure 2:**
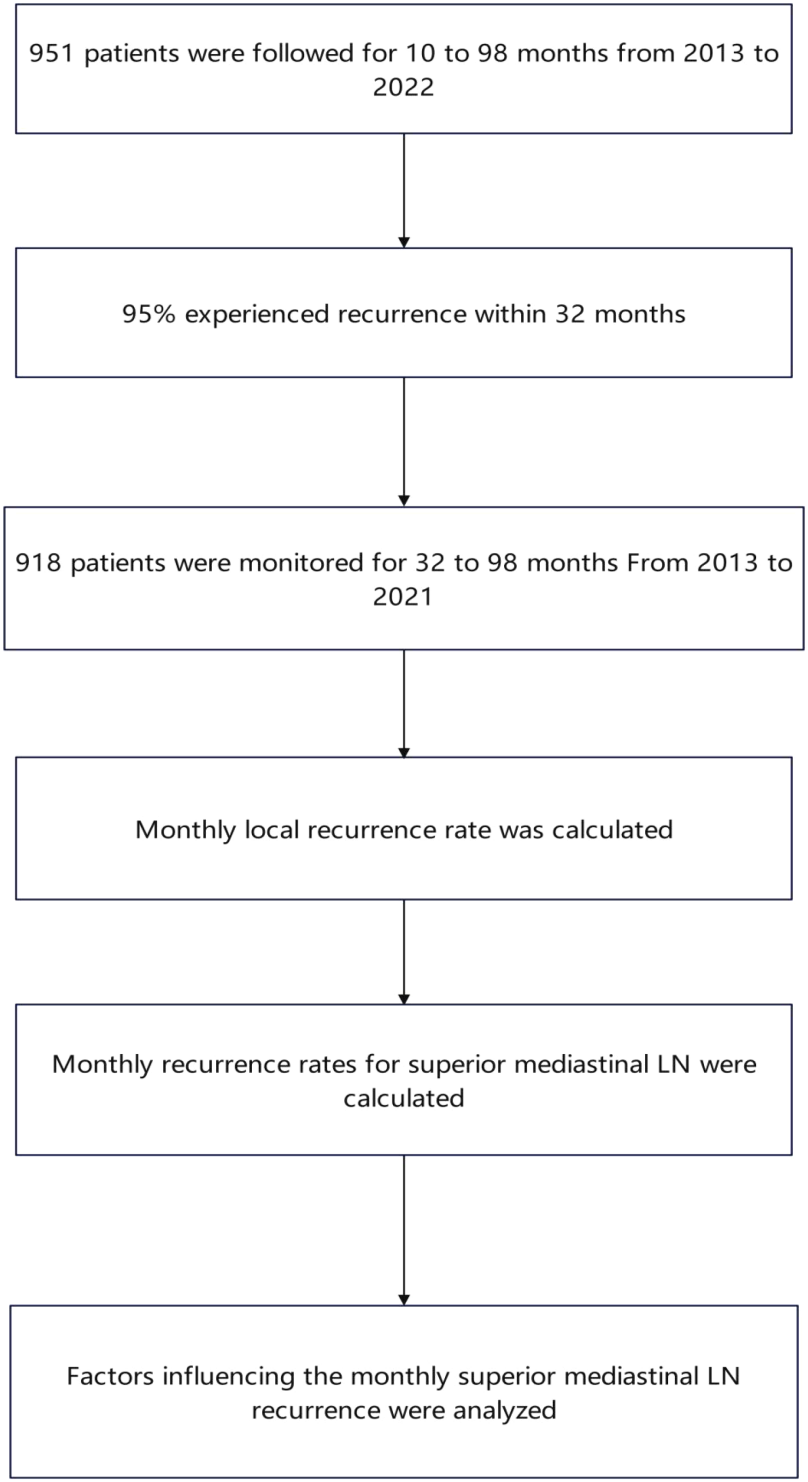
Flow chart: A total of 918 consecutive patients with esophageal carcinoma underwent curative-intent surgery at Jining First People’s Hospital between January 2013 and May 2021 and were prospectively monitored for 32–98 months.

**Figure 3:**
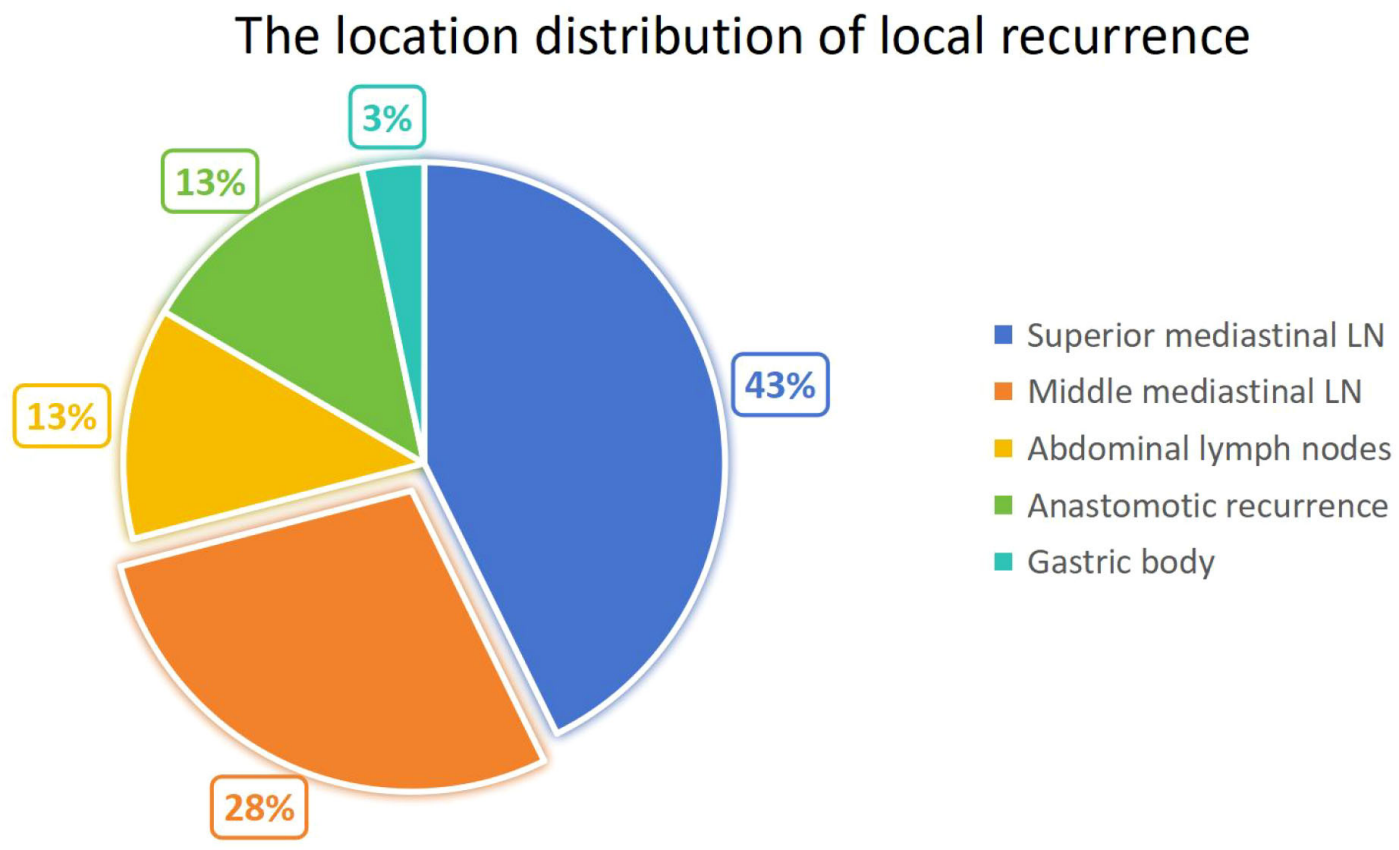
The probabilities of local recurrence among patients were as follows: upper mediastinal lymph node recurrence (43%), middle mediastinal lymph node recurrence (28%), abdominal lymph node recurrence (13%), anastomotic recurrence (13%), and gastric body recurrence (3%).

A total of 918 consecutive patients with esophageal carcinoma underwent curative-intent surgery at Jining First People’s Hospital between January 2013 and May 2021 and were prospectively monitored for 32–98 months (**Figure 2**). By study closure, 224 (24.4%) had developed local recurrence. The upper mediastinum—comprising the bilateral recurrent laryngeal and upper para-esophageal nodes—accounted for the majority of these events (103 cases, 46.0%), followed by the middle mediastinum (middle para-esophageal, subcarinal and main-pulmonary-window nodes; 68 cases, 30.4%) and the peri-celiac region (nodes along the left gastric, common hepatic and splenic arteries, lesser gastric curvature and pericardial area; 30 cases, 13.4%). Anastomotic and gastric-conduit recurrences were identified in 32 (14.3%) and 8 (3.6%) patients, respectively (**Figure 3**).

### Temporal trend of recurrence

To delineate the evolving pattern of recurrence after esophageal squamous cell carcinoma (ESCC) surgery over the past decade, we calculated the monthly incidence of postoperative relapse.

**Figure 4:**
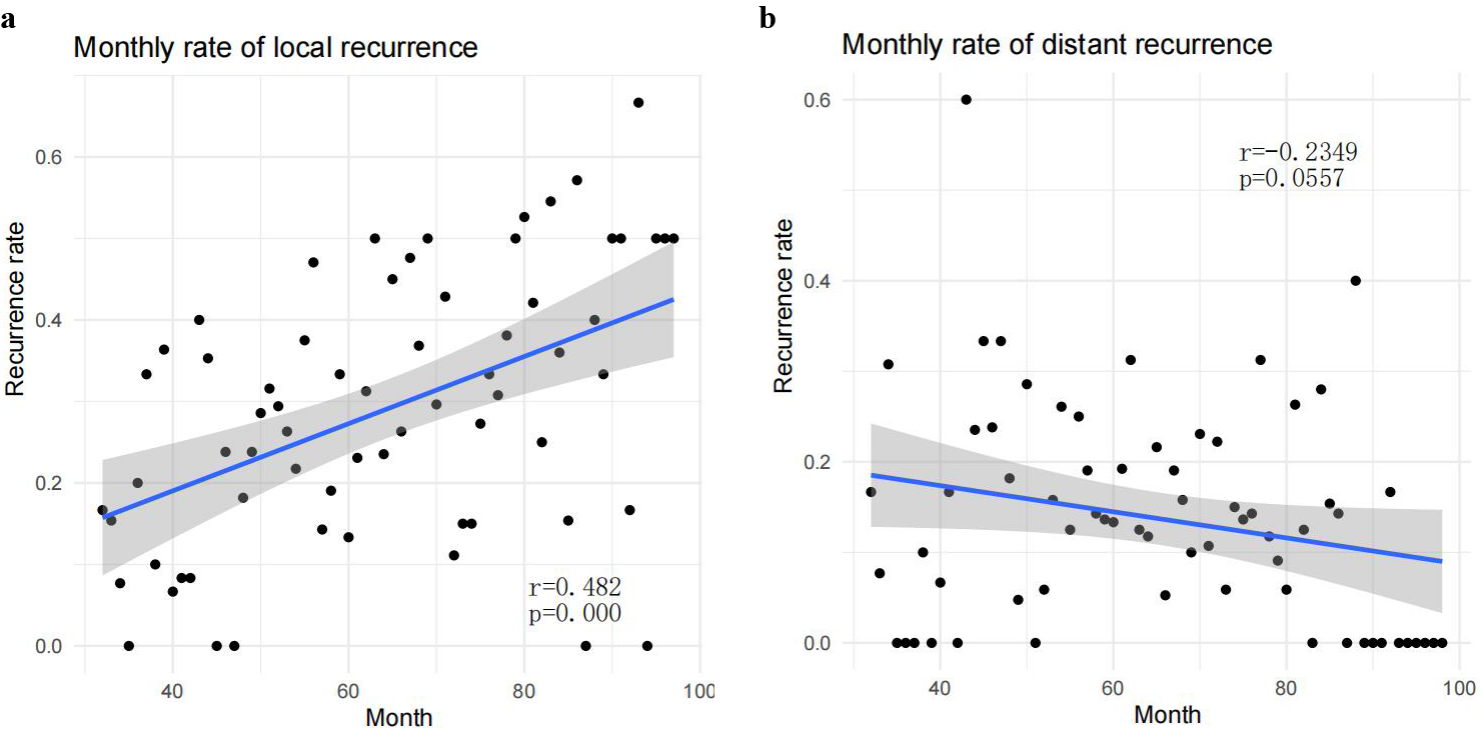
**(A)** The local monthly recurrence rate of esophageal cancer after surgery showed a significant year-over-year decreasing trend (r = 0.482, p = 0.000). **(B)** No significant downward trend was observed in the distant monthly recurrence rate of esophageal cancer after surgery (r = -0.2349, p = 0.0557).

Local recurrence exhibited a pronounced and statistically significant year-on-year decline (p < 0.001) with a moderate positive temporal correlation (r = 0.482, **Figure 4A**). Conversely, the incidence of distant metastasis showed no significant downward trend and lacked statistical correlation with time (**Figure 4B**).While not a primary focus of this temporal analysis, the potential impact of evolving neoadjuvant regimens, including emerging immunochemotherapy protocols, on recurrence patterns warrants further investigation.

### Stratified analysis of local recurrence

Although our temporal analysis demonstrated a progressive decline in overall local recurrence after esophagectomy, the specific anatomic sites driving this trend remained undefined. We therefore stratified local recurrences into six compartments: upper mediastinal lymph nodes (UMLNs), middle mediastinal lymph nodes, abdominal lymph nodes, anastomotic sites, gastric conduit, and resection margins.

**Figure 5:**
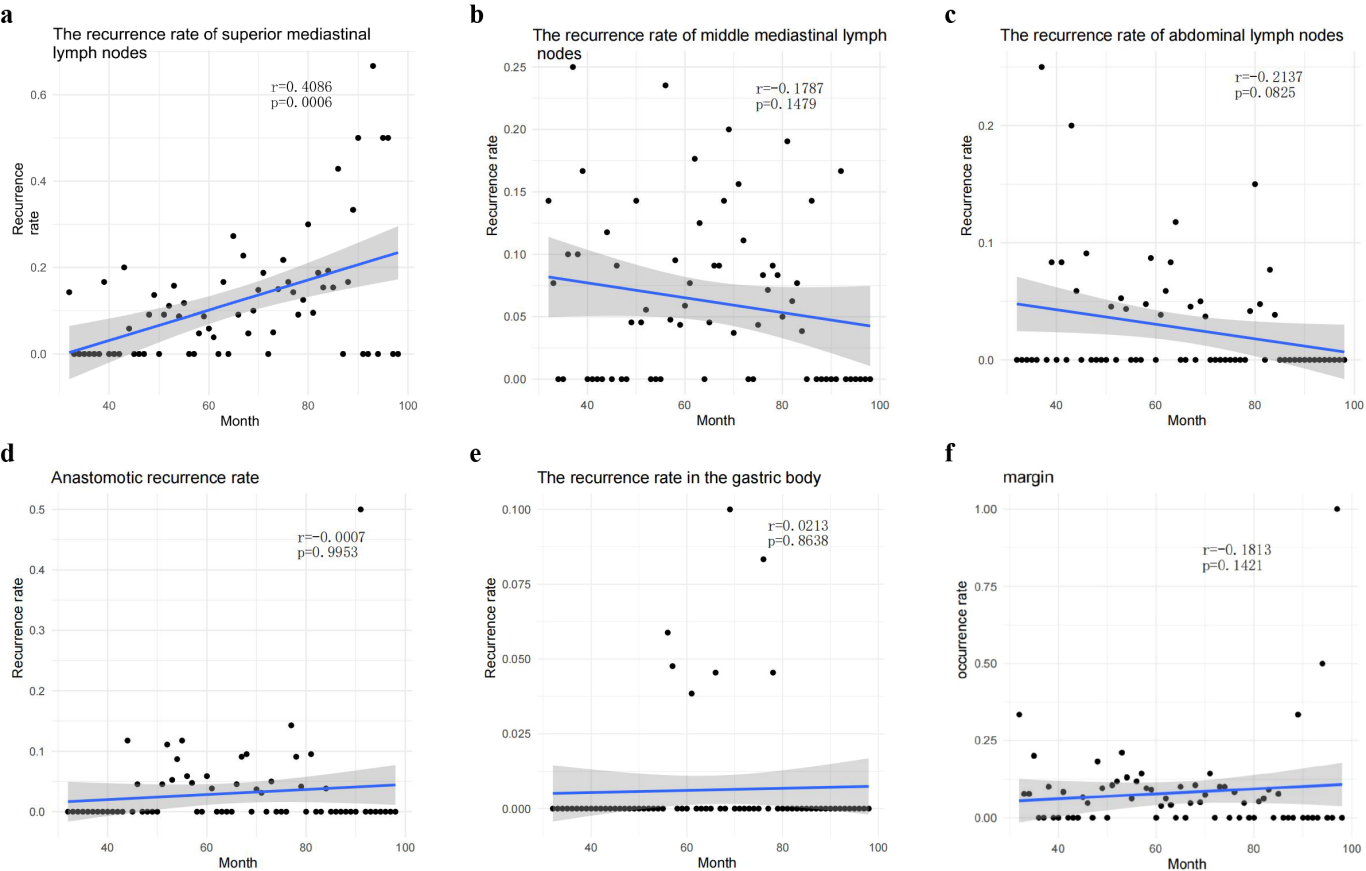
Only the recurrence rate of upper mediastinal lymph nodes showed a significant month-over-month decline(r = 0.4086, p = 0.0006), as shown in **(A)**. No such trends were observed in other local recurrence sites, including middle mediastinal lymph nodes, abdominal lymph nodes, anastomotic sites, the gastric body, and margin, as illustrated in figures **(B–F)**.

Among these, only the monthly incidence of UMLN recurrence exhibited a consistent year-on-year decrease (**Figure 5**). Correlation analysis confirmed a significant inverse temporal association (r = –0.409, p < 0.001). In contrast, recurrence rates within the other five compartments remained stable over the study period. These data indicate that the observed reduction in overall local recurrence during the past decade is attributable almost exclusively to the sustained decline in UMLN recurrence.

### Univariate and multivariate analyses of UMLNs recurrence

Based on the recurrence status of the superior mediastinal lymph nodes, the subjects were categorized into a recurrence group and a non-recurrence group, followed by univariate and multivariate analyses. The univariate analysis indicated that the number of lymph nodes dissected (P = 0.008), postoperative therapy (P = 0.038), sex (P = 0.033), alcohol consumption (P = 0.003), surgical method (P = 0.001), and pTNM stage (P = 0.003) were independent factors influencing the recurrence of superior mediastinal lymph nodes, as shown in **Table 1**. The COX multivariate analysis revealed that alcohol consumption (P = 0.021), tumor location (P = 0.01), surgical method (P = 0.000), surgical margin (P = 0.044), number of lymph nodes dissected (P = 0.001), pTNM stage (P = 0.000), and postoperative therapy (P = 0.024) were significant factors affecting recurrence, as shown in **Table 2**.

**Table 1:** Clinical characteristics and univariate analysis of upper mediastinal lymph node recurrence.

Variable	Overall, N = 918^1^	Recurrence		P-value^2^
		No N = 815 (89%)^1^	Yes N = 103 (11%)^1^	
age	66.00 [61.00, 70.00]	66.00 [61.00, 71.00]	66.00 [59.00, 69.00]	0.127
Number of lymph nodes dissected	19.00 [14.00, 25.00]	19.00 [14.00, 25.00]	18.00 [11.00, 22.50]	0.008
Postoperative therapy				0.038
None	520 (56.64%)	471 (57.79%)	49 (47.57%)	
Chemotherapy	209 (22.77%)	174 (21.35%)	35 (33.98%)	
Radiotherapy	55 (5.99%)	49 (6.01%)	6 (5.83%)	
Chemoradiotherapy	134 (14.60%)	121 (14.85%)	13 (12.62%)	
Sex				0.033
Female	215 (23.42%)	200 (24.54%)	15 (14.56%)	
Male	703 (76.58%)	615 (75.46%)	88 (85.44%)	
Smoke				0.208
No	369 (40.20%)	334 (40.98%)	35 (33.98%)	
Yes	549 (59.80%)	481 (59.02%)	68 (66.02%)	
Drink				0.003
No	557 (60.68%)	509 (62.45%)	48 (46.60%)	
Yes	361 (39.32%)	306 (37.55%)	55 (53.40%)	
Tumor location				0.717
Upper	104 (11.33%)	94 (11.53%)	10 (9.71%)	
Middle	544 (59.26%)	480 (58.90%)	64 (62.14%)	
Lower	184 (20.04%)	162 (19.88%)	22 (21.36%)	
Abdominal	86 (9.37%)	79 (9.69%)	7 (6.80%)	
Neoadjuvant therapy				0.416
No	883 (96.19%)	782 (95.95%)	101 (98.06%)	
Yes	35 (3.81%)	33 (4.05%)	2 (1.94%)	
Surgery method				<0.001
Left thoracotomy	371 (40.41%)	306 (37.55%)	65 (63.11%)	
Ivor lewis	16 (1.74%)	13 (1.60%)	3 (2.91%)	
MIE	507 (55.23%)	475 (58.28%)	32 (31.07%)	
Mediastinoscope	24 (2.61%)	21 (2.58%)	3 (2.91%)	
Differentiation				0.183
Low	255 (27.78%)	222 (27.24%)	33 (32.04%)	
Moderate	410 (44.66%)	361 (44.29%)	49 (47.57%)	
High	182 (19.83%)	164 (20.12%)	18 (17.48%)	
Unknow	71 (7.73%)	68 (8.34%)	3 (2.91%)	
Margin				0.076
No	854 (93.03%)	763 (93.62%)	91 (88.35%)	
Yes	64 (6.97%)	52 (6.38%)	12 (11.65%)	
PTNM				0.003
Tis	18 (1.96%)	18 (2.21%)	0 (0.00%)	
IA	62 (6.75%)	61 (7.48%)	1 (0.97%)	
IB	44 (4.79%)	41 (5.03%)	3 (2.91%)	
IIA	124 (13.51%)	115 (14.11%)	9 (8.74%)	
IIB	232 (25.27%)	207 (25.40%)	25 (24.27%)	
IIIA	44 (4.79%)	40 (4.91%)	4 (3.88%)	
IIIB	323 (35.19%)	276 (33.87%)	47 (45.63%)	
IVA	71 (7.73%)	57 (6.99%)	14 (13.59%)	

^1^Median [IQR]; n (%).

^2^Wilcoxon rank sum test; Pearson’s Chi-squared test; Fisher’s exact test; Fisher’s Exact Test for Count Data with simulated p-value (based on 2000 replicates).

Note: The univariate analysis indicated that the number of lymph nodes dissected (P = 0.008), postoperative therapy (P = 0.038), sex (P = 0.033), alcohol consumption (P = 0.003), surgical method (P = 0.001), and pTNM stage (P = 0.003) were independent factors influencing the recurrence of superior mediastinal lymph nodes.

**Table 2:** COX multivariate analysis of factors influencing superior mediastinal lymph node recurrence.

Factor	P value	Exp(B)	95.0% Exp(B) CI	
			Lower limit	Upper limit
Sex	0.511	0.799	0.41	1.558
Age	0.136	0.981	0.956	1.006
Smoke	0.752	1.088	0.646	1.832
Drink	0.021	0.571	0.354	0.92
Tumor location	0.010			
upper	0.001	5.65	1.959	16.291
middle	0.004	3.284	1.46	7.387
lower	0.036	2.537	1.063	6.055
Neoadjuvant therapy	0.471	1.732	0.389	7.708
Surgery method	0.000			
Left thoracotomy	0.643	1.339	0.39	4.601
Ivor lewis	0.983	1.019	0.187	5.552
MIE	0.171	0.413	0.116	1.465
Differentiation	0.994			
low	0.812	1.166	0.327	4.158
Moderate	0.865	1.114	0.32	3.879
high	0.864	1.121	0.305	4.119
Margin	0.044	0.525	0.28	0.983
Number of lymph nodes dissected	0.001	0.952	0.925	0.979
PTNM	0.000			
Tis	0.960	0	0	2.92E+224
IA	0.000	0.023	0.003	0.192
IB	0.002	0.112	0.028	0.438
IIA	0.000	0.12	0.048	0.298
IIB	0.000	0.217	0.105	0.448
IIIA	0.040	0.298	0.094	0.948
IIIB	0.016	0.457	0.241	0.866
Postoperative therapy	0.024			
None	0.039	1.985	1.035	3.809
Chemotherapy	0.004	2.69	1.383	5.233
Radiotherapy	0.613	1.291	0.48	3.476

The COX multivariate analysis identified several significant factors affecting recurrence, including alcohol consumption (P = 0.021), tumor location (P = 0.010), surgical method (P < 0.001), surgical margin (P = 0.044), number of lymph nodes dissected (P < 0.001), pTNM stage (P < 0.001), and postoperative therapy (P = 0.024).

### Multivariable analysis of monthly UML recurrence

To isolate the determinants underlying the observed decline in upper-mediastinal lymph-node (UML) relapse, we performed a time-series multivariable analysis of monthly recurrence rates. After adjustment for potential confounders, advanced pathologic stage (≥III) and a minimally invasive esophagectomy (MIE) approach emerged as independent predictors of the temporal reduction in UML recurrence (**Table 3**).

**Table 3:** Multivariate analysis of the monthly recurrence rate.

Factor	P value	Exp(B)	95.0% Exp(B) CI	
			Lower limit	Upper limit
Drink	0.0664	-0.1682	-0.348	0.0117
Postoperative therapy including radiotherapy	0.2828	-0.1112	-0.348	0.094
Upper or middle tumor	0.9548	-0.0058	-0.2098	0.1982
MIE Surgery method*	0.0023	-0.2176	-0.354	-0.0812
Number of lymph nodes dissected >20	0.6172	-0.0338	-0.1682	0.1007
PTNM stage ≥III	0.0237	0.1936	0.3604	0.0237

**Table 3** The analysis of time series data revealed that Ptnm and surgical method were independent factors influencing the monthly recurrence rate of the upper mediastinum.

*MIE: minimally invasive esophagectomy.

### Correlation analysis of monthly UML recurrence

**Figure 6:**
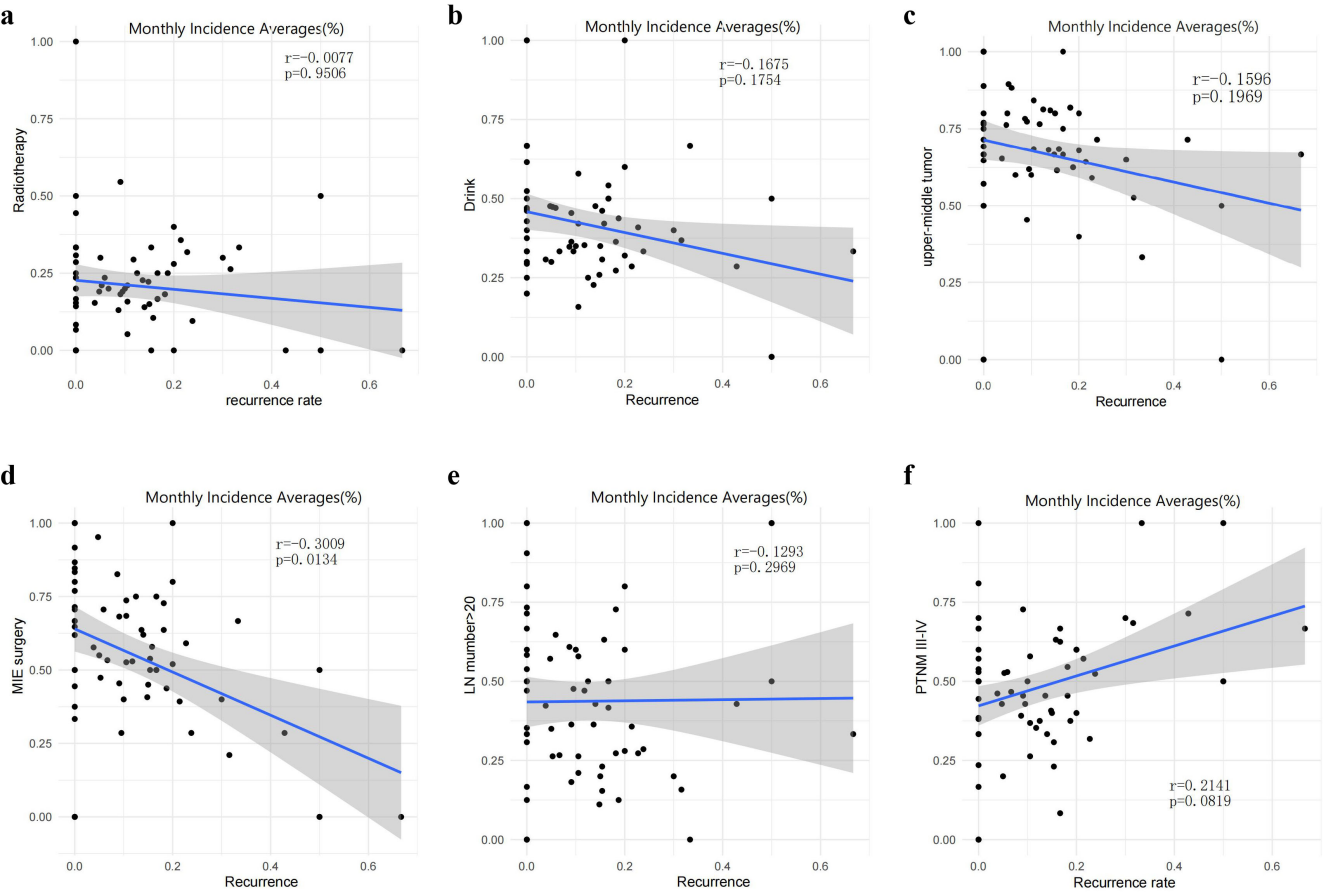
Correlation analysis between monthly recurrence rate and occurrence rate of each factor. Only the MIE surgical method demonstrated a significant negative correlation with the monthly average recurrence rate (r = -0.3009, p = 0.0134), illustrated in **(D)**. No correlation was observed between the postoperative adjuvant therapies, including radiotherapy, alcohol consumption, tumor location in the upper and middle segments, lymph node dissection (>20), stage III-IV, and the monthly recurrence rate, as shown in **(A–F)**.

To corroborate the multivariable findings, we examined the bivariate relationships between candidate variables and the monthly rate of upper-mediastinal lymph-node recurrence. Only the adoption of a minimally invasive esophagectomy (MIE) exhibited a significant inverse correlation (r = –0.301, p = 0.013; **Figures 6D–F**). In contrast, neither postoperative radiotherapy, alcohol consumption, tumor location in the upper or middle third, extent of lymphadenectomy (>20 nodes), nor advanced stage (III–IV) demonstrated any appreciable association with temporal changes in recurrence (**Figure 6**).

## Discussion

Surgery remains an indispensable component in the treatment of esophageal cancer (3). However, the postoperative recurrence rate is alarmingly high, ranging from 45% to 55%, with lymph nodes being the most frequently involved sites (13–15). Specifically, cervical lymph nodes(19%), abdominal lymph nodes (17%), and upper mediastinal lymph nodes(17%) are the most commonly affected (16). Recurrences of metastatic lymph nodes, particularly in the upper mediastinum and cervical regions, predominantly occur in the upper esophagus (17). According to the eighth edition of the AJCC guidelines, metastatic involvement of abdominal lymph nodes is primarily seen in lower esophageal cancer cases. Additionally, distant metastasis poses a significant challenge, with postoperative rates reported between 20.9% and 27% (18, 19). In our cohort of 918 patients, the local recurrence rate was 24.4%, while the distant recurrence rate stood at 15.7%. Analyzing recurrence rates on a monthly basis, we observed a downward trend in local recurrence over the past decade, although the distant recurrence rate remained relatively stable. Further analysis revealed a significant decline in the recurrence of upper mediastinal lymph nodes, while the recurrence rates for abdominal lymph nodes, mid-mediastinal lymph nodes, anastomosis sites, and the gastric body did not show significant trends.

Time series analysis revealed a progressive decline in local recurrence rates of esophageal cancer over the past 10 years, with a pronounced reduction in metastases to the middle and upper mediastinal lymph nodes. Preliminary multivariate analysis demonstrated a significant association between upper mediastinal lymph node recurrence and key variables including surgical approach, alcohol consumption, lymphadenectomy extent, and pathological TNM stage. Temporal analysis further identified both pathological stage (pTNM ≥III) and surgical technique as independent risk factors influencing the monthly recurrence rate within the upper mediastinum.

This trend coincides with two pivotal advancements in esophageal cancer management. First, the systematic implementation of endoscopic screening programs has shifted the clinical presentation toward earlier-stage disease, enabling curative resection prior to locoregional dissemination. Second, refinements in minimally invasive techniques—particularly the widespread adoption of minimally invasive esophagectomy (MIE)—have permitted more anatomically precise and comprehensive lymphadenectomy, even within technically challenging upper mediastinal compartments (20, 21). The synergistic effects of earlier detection (facilitating timely intervention) and optimized surgical resection (enhancing regional disease control) collectively underpin the observed attenuation in local recurrence risk.

Despite our compelling findings, this study has several limitations that should be acknowledged. Firstly, its retrospective, single-center nature introduces potential for unmeasured confounding and selection bias, despite multivariate adjustment for known variables. Consequently, the observed association between minimally invasive esophagectomy and reduced recurrence should be interpreted as indicative of a temporal trend rather than definitive causation. Secondly, the variable ‘change in surgical approach’ represents a complex evolution in technique and expertise over time, making it difficult to isolate the precise contributory element. Furthermore, differences in follow-up duration between earlier and later cohorts potentially introduce lead-time bias, which may overestimate the magnitude of the observed benefit. Finally, the findings from our high-volume tertiary center may not be fully generalizable to all clinical settings due to the significant learning curve associated with advanced surgical techniques.

In summary, this study delineates a sustained, time-dependent decrease in postoperative recurrence for resectable esophageal cancer, with the most pronounced attenuation observed in the upper mediastinal nodal basin. Temporal risk modelling identifies pathological stage (pTNM ≥ III) as an independent determinant of monthly recurrence hazard within this anatomic compartment. Importantly, the progressive transition toward minimally invasive esophagectomy (MIE) is strongly and independently associated with a reduced probability of recurrence at these critical stations. Beyond the benefits of MIE per se, the incremental value of an extended, three-field lymphadenectomy is evident in further suppressing cervical nodal relapse (22, 23). Finally, emerging immunoadjuvant strategies—exemplified by the regimen evaluated in the chemat577 trial (24)—constitute a promising therapeutic frontier that may consolidate and extend the oncological gains achieved through refined surgical technique.

## Conclusions

The study demonstrated a consistent decline in local recurrence rates over the past decade, particularly in upper mediastinal lymph nodes. The adoption of minimally invasive surgical techniques and improvements in Neoadjuvant management likely contributed to this trend. Alcohol consumption was identified as a risk factor for upper mediastinal recurrence. Future research should focus on optimizing neoadjuvant strategies, including immunochemotherapy, and exploring the potential benefits of more extensive lymph node dissection and postoperative immunotherapy to further reduce recurrence rates.

## Data Availability

The raw data supporting the conclusions of this article will be made available by the authors, without undue reservation.
